# Supplementary material for: Regulation of Antimicrobial Effect of Hemicyanine-Based Photosensitizer via Supramolecular Assembly
Source: Nanomaterials (Basel). 2022 Aug 24;12(17):2905. doi: 10.3390/nano12172905 (PMC9457667; doi:10.3390/nano12172905)
Supplement: Supplementary file 1 [file nanomaterials-12-02905-s001.zip › nanomaterials-1860037-supplementary.pdf]

nanomaterials

Supplementary material

Regulation of Antimicrobial Effect of Hemicyanine-based photosensitizer via Supramolecular Assembly

Huanxiang Yuan [1], Shaochuan Jia [1], Zelin Li [1], Jian Liu [2], Xiaoyu Wang [3,*] and Ruilian Qi [1,*]

1 Department of Chemistry, College of Chemistry and Materials Engineering, Beijing Technology and Business University, Beijing 100048, China
2 Institute of Chemistry, Chinese Academy of Sciences, Beijing 100090, China
3 School of Materials Science and Engineering, University of Science and Technology Beijing, Beijing 100083, China
* Correspondence: wangxy@ustb.edu.cn (X.W.); qiruilian@iccas.ac.cn (R.Q.)

Materials and Measurements

All chemicals were purchased from the commercial sources (Sigma-Aldrich Shanghai China, and Alfa-Aesar Shanghai China). All organic solvents were obtained from Beijing Chemical Works. UV-Vis absorption spectra were got from a JASCO V-550 spectrophotometer. Fluorescence spectra were measured on a Hitachi F-4500 fluorometer. Three microorganisms (Amp^r *E. coli*, *S. aureus*, *C. albicans*) were selected as representative strains, which were purchased from China General Microbiological Culture Collection Center. The concentration of microbial suspensions (OD_{600}) tested by a UV-Vis spectrophotometer (JASCO V-550) at 600 nm. Bacterial culture related consumables are used after sterilization. Photographs of bright-field and fluorescence-field were taken with a fluorescence microscope (Ti2-U, Nikon, Tokyo, Japan). Zeta potentials were measured on a Nano ZS (ZEN3600) (Malvern, Malvern, UK) system. The ^{1}H NMR spectra were collected by Bruker Avance III 400 HD nuclear magnetic resonance spectrometer.

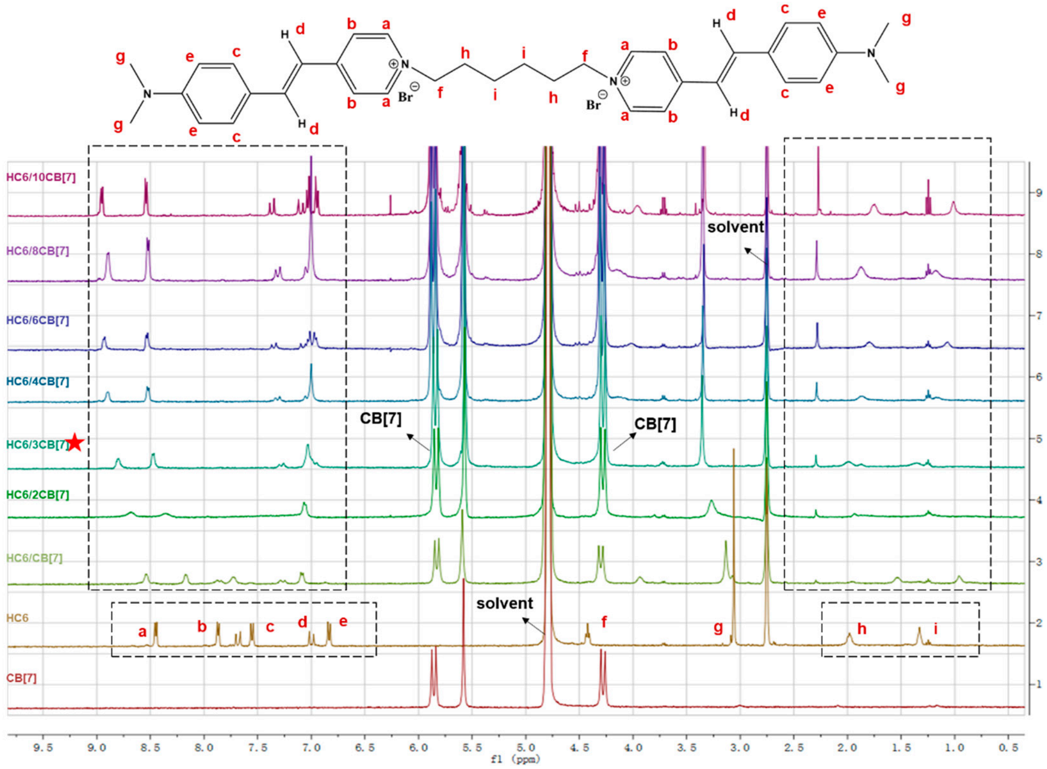

Figure S1. ^{1}H NMR (400MHz, D_2O with 0.17% DMSO-d_6) spectra of HC6 before and after adding CB[7]. The chemical shifts of protons in aromatic and dimethyl amino groups gradually move to downfield, indicating the assembly complexes HC6/CB[7] form. This is because that the formation of HC6/CB[7] complexes can reduce the aggregation degree of HC6, resulting in the decrease of electron density in aromatic and dimethyl amino groups which lead to a higher ppm value in NMR spectra.

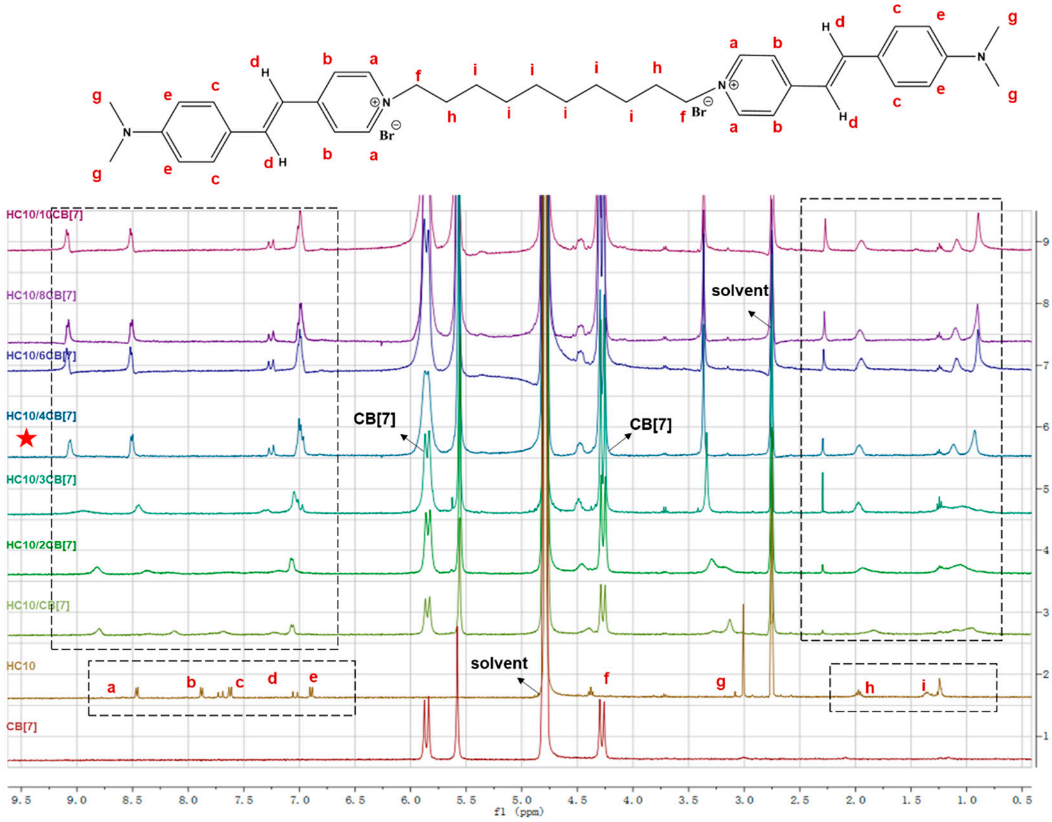

.

Figure S2. ^{1}H NMR (400MHz, D_2O with 0.17% DMSO-d_6) spectra of HC10 before and after adding CB[7].

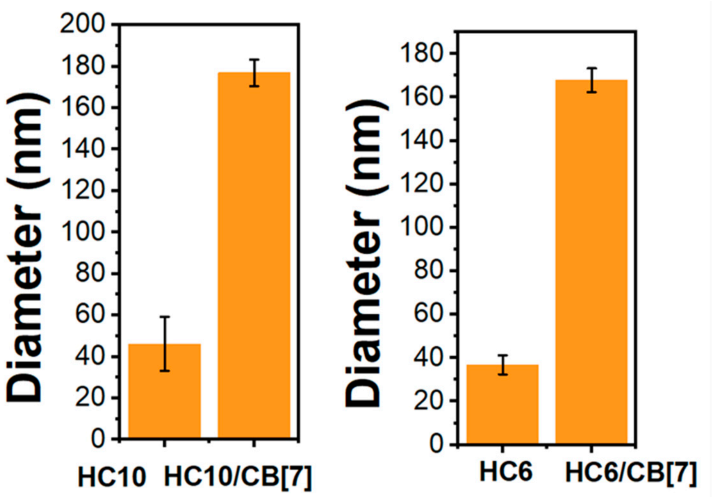

.

Figure S3. Size histograms of HC6 or HC10 and its supramolecular complex HCs/CB[7].

Table S1. Zeta potential data of Amp[r] *E. coli*, *S. aureus* and *C. albicans* before and after binding with HCs or supramolecular conjugate HCs/CB[7] complex, respectively.

**Samples**		**Zeta potentials (ζ, mV)**
Amp[r] *E. coli*	Blank	-44.5 ± 0.2
	HC6	-41.6 ± 0.4
	HC6/CB[7]	-45.4 ± 0.3
	HC10	-39.1 ± 0.5
	HC10/CB[7]	-44.5 ± 0.1
S. aureus	Blank	-26.2 ± 0.1
	HC6	-23.4 ± 0.4
	HC6/CB[7]	-24.1 ± 0.1
	HC10	-19.2 ± 1.0
	HC10/CB[7]	-26.1 ± 0.6
C. albicans	Blank	-18.0 ± 0.1
	HC6	-17.1 ± 0.3
	HC6/CB[7]	-17.3 ± 0.3
	HC10	-18.2 ± 0.9
	HC10/CB[7]	-18.3 ± 1.1

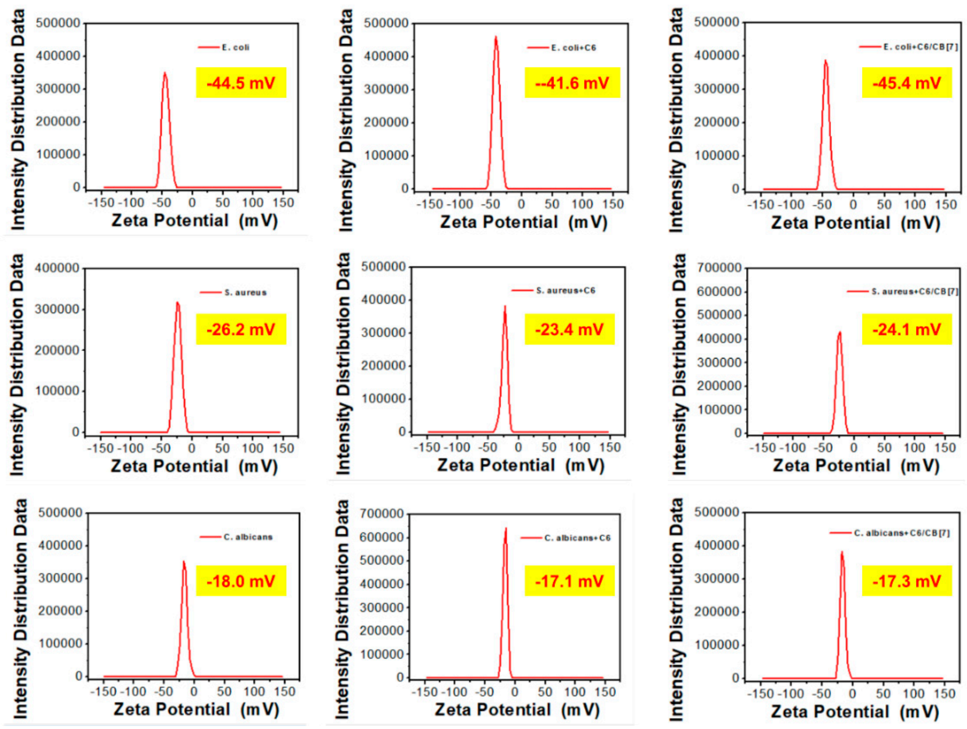

Figure S4. Zeta potentials of the three pathogens before and after binding with HC6 or supramolecular conjugate complex HC6/CB[7].

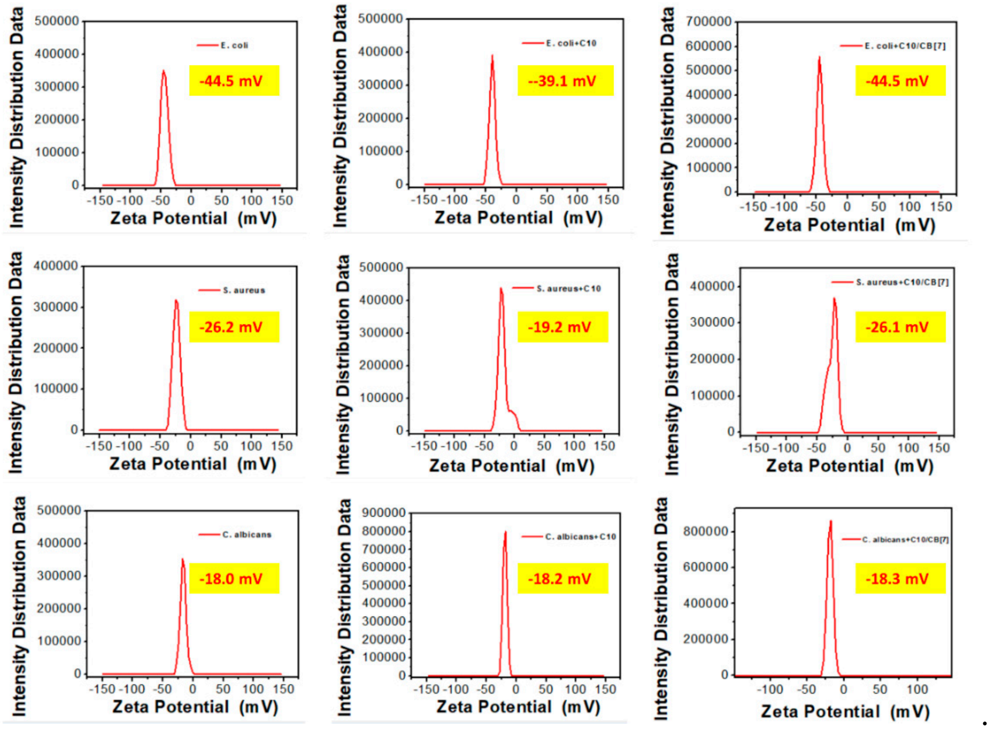

Figure S5. Zeta potentials of the three pathogens before and after binding with HC10 or supramolecular conjugate complex HC10/CB[7].

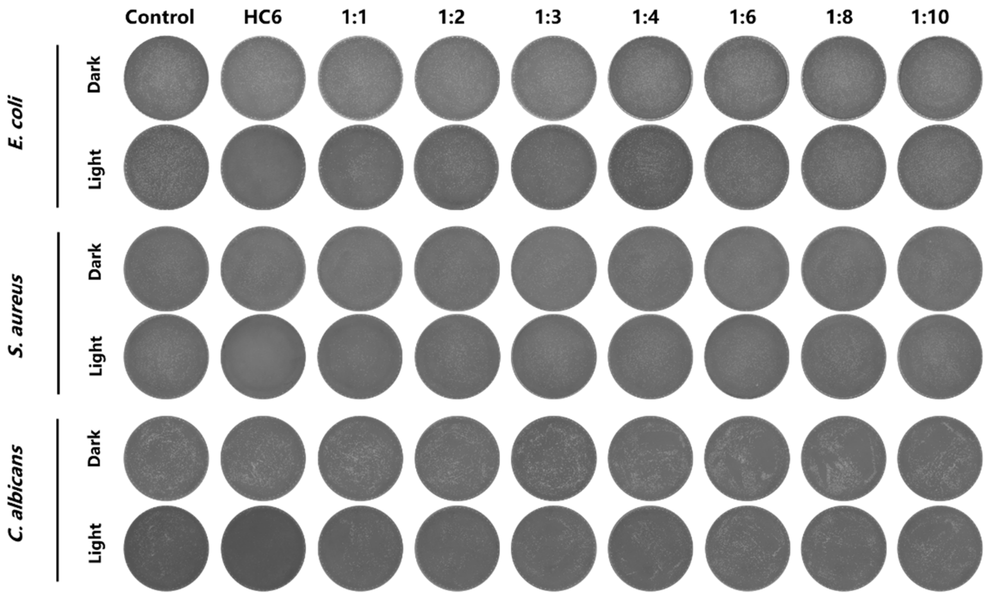

Figure S6. Plate photographs of *E. coli*, *S. aureus* and *C. albicans* acting with HC6 and its supramolecular assemblers in different proportions. [HC6] = 25 μM, [CB[7]] = 0, 25, 50, 75, 100, 150, 200, 250 μM.

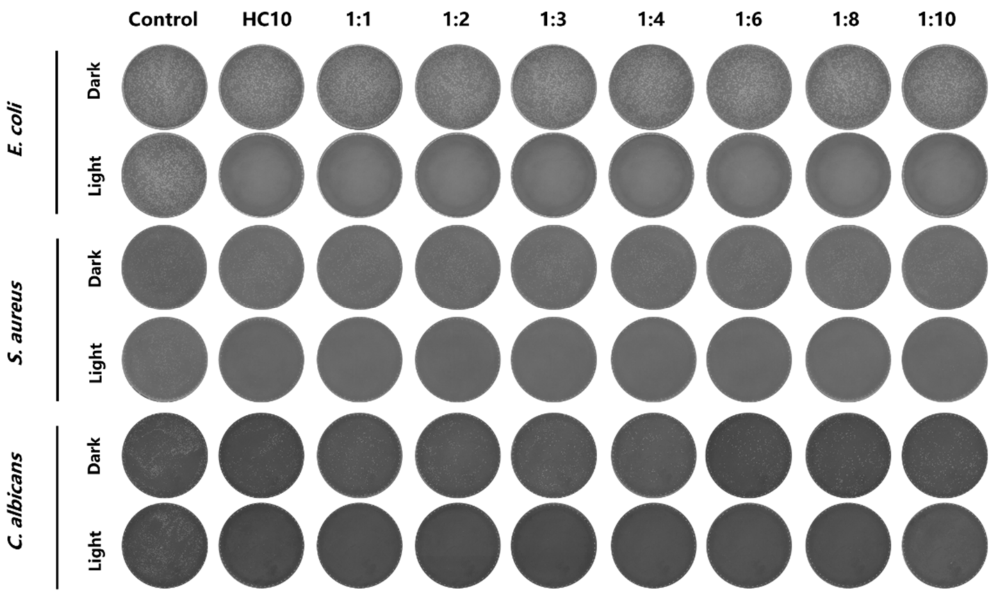

Figure S7. Plate photographs of *E. coli*, *S. aureus* and *C. albicans* acting with HC10 and its supramolecular assemblers in different proportions. [HC10] = 25 μM, [CB[7]] =0, 25, 50, 75, 100, 150, 200, 250 μM.

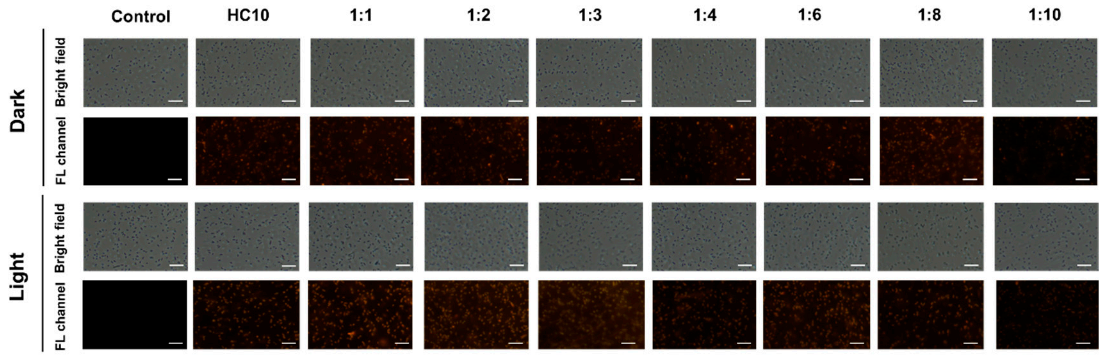

.

Figure S8. Fluorescence microscope images of *E. coli* before and after binding with HC10 and HC10/CB[7] in different proportions under light (65 mW/cm^2) and darkness, [HC10] = 25 μM, [CB[7] = 0, 25, 50, 75, 100, 150, 200, 250 μM, the scale is 20 μm.

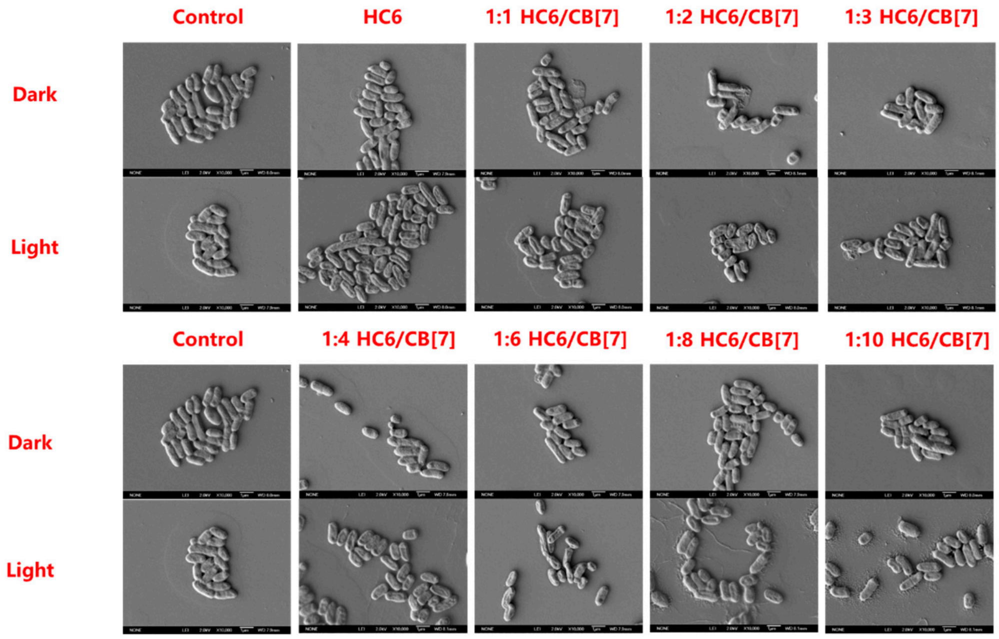

Figure S9. SEM images of Amp^r *E. coli* with HC6 and HC6/CB[7] before and after the interaction under dark or light (65 mW/cm^2), the scale is 1 µm.
